# Competitive endogenous network of circRNA, lncRNA, and miRNA in osteosarcoma chemoresistance

**DOI:** 10.1186/s40001-023-01309-x

**Published:** 2023-09-16

**Authors:** Shuang Qin, Yuting Wang, Chunhui Ma, Qi Lv

**Affiliations:** 1 Department of Medical Imaging, Tongji Hospital, Tongji University School of Medicine, Xincun Road No. 389, Shanghai, 200065 China; 2 Department of Orthopedics, Shanghai General Hospital of Shanghai Jiaotong University, Wujin Road No. 85, Shanghai, 200080 China

**Keywords:** Osteosarcoma, CircRNA, LncRNA, miRNA, Competitive endogenous RNA, Chemotherapy resistance, Multidrug resistance

## Abstract

Osteosarcoma is the most prevalent and fatal type of bone tumor. Despite advancements in the treatment of other cancers, overall survival rates for patients with osteosarcoma have stagnated over the past four decades Multiple-drug resistance—the capacity of cancer cells to become simultaneously resistant to multiple drugs—remains a significant obstacle to effective chemotherapy. The recent studies have shown that noncoding RNAs can regulate the expression of target genes. It has been proposed that “competing endogenous RNA” activity forms a large-scale regulatory network across the transcriptome, playing important roles in pathological conditions such as cancer. Numerous studies have highlighted that circular RNAs (circRNAs) and long noncoding RNAs (lncRNAs) can bind to microRNA (miRNA) sites as competitive endogenous RNAs, thereby affecting and regulating the expression of mRNAs and target genes. These circRNA/lncRNA-associated competitive endogenous RNAs are hypothesized to play significant roles in cancer initiation and progression. Noncoding RNAs (ncRNAs) play an important role in tumor resistance to chemotherapy. However, the molecular mechanisms of the lncRNA/circRNA-miRNA-mRNA competitive endogenous RNA network in drug resistance of osteosarcoma remain unclear. An in-depth study of the molecular mechanisms of drug resistance in osteosarcoma and the elucidation of effective intervention targets are of great significance for improving the overall recovery of patients with osteosarcoma. This review focuses on the molecular mechanisms underlying chemotherapy resistance in osteosarcoma in circRNA-, lncRNA-, and miRNA-mediated competitive endogenous networks.

## Introduction

Osteosarcoma (OS) is a primary malignant bone tumor, more commonly observed in children and adolescents [1]. In the 1970s, amputation stood as the standard OS treatment, yielding a 5-year survival rate of only 20% [2, 3]. The emergence of chemotherapy agents [4] has elevated the post-treatment 5-year OS survival rate [5–8]. However, long-term chemotherapy poses the risk that the patient’s cells will develop resistance to the chemotherapeutic drugs, eventually, eventually culminating in OS recurrence, distant metastasis, and treatment failure.

The resistance of OS to therapy is intimately linked with multidrug resistance, which emanates from prolonged exposure of cancerous cells to a particular chemotherapeutic agent. This exposure can lead to cross-resistance against diverse chemotherapeutic agents with varying structures and functions. The effectiveness of chemotherapy in OS is markedly impacted by multidrug resistance. Presently, there exist no conventional methods to surmount chemotherapy resistance in malignancies without inducing adverse side effects. The exploration of novel generations of antitumor medications to combat tumor resistance has become a pivotal concept in the realm of cancer therapy.

For the purpose of curtailing OS recurrence and metastasis rates, it becomes of paramount significance to elucidate the manifold resistance mechanisms of OS against chemotherapeutic drugs and to investigate potential strategies for reversing this process. This review delves into the intricate mechanisms of drug resistance in OS, with particular emphasis on circRNA-, lncRNA-, and miRNA-mediated competitive endogenous networks.

## Noncoding RNAs

It has been demonstrated that noncoding RNAs (ncRNAs), such as circular RNAs (circRNAs), long noncoding RNAs (lncRNAs), and microRNAs (miRNAs), play significant roles in the regulation of cancer biology. The primary function of ncRNAs, which are not transcribed into proteins, is to regulate gene expression. Numerous biological properties of ncRNAs have been identified over the past few years. In addition, a growing number of ncRNAs is thought to play roles in OS tumorigenesis, invasion, metastatic progression, apoptosis, and drug resistance [9]. Salmena first proposed the competitive endogenous RNA hypothesis in 2011 [10], suggesting that lncRNA might regulate the expression of downstream genes by competitively binding to miRNA through microRNA response elements. Competitive endogenous RNA is not a novel ribonucleic acid molecule; instead, it is a novel mechanism for factor regulation. There is mounting evidence that noncoding RNAs, particularly circRNAs, lncRNAs, and miRNAs, form a competitive endogenous RNA-restrictive network with mRNAs, and this network influences drug resistance [11–14]. Importantly, noncoding RNAs play a role in OS drug resistance due to their competitive endogenous RNA mechanisms.

## circRNA mediated competitive endogenous RNA in OS chemoresistance

Since their discovery in 1976, circRNAs have been hypothesized to result from incorrect shearing and low expression levels [15, 16]. Numerous studies have demonstrated that circRNAs are involved in various pathophysiological processes in the body and are abnormally expressed in a wide range of malignant tumors, including OS [22], gastric cancer [17], bladder cancer [18], liver cancer [19], colorectal cancer [20], and breast cancer [21]. Tumorigenesis and modifications in the biological functions of cells result from abnormal circRNA expression [23, 24]. In addition, it has been demonstrated that circRNAs are associated with tumor therapy resistance; for example, circ_0026359 promotes cisplatin resistance in stomach cancer [25]. The results of several studies have shown that circRNAs have a significant impact on chemotherapy resistance in OS (Table 1).

**Table 1:** circRNAs and osteosarcoma chemotherapy resistance

Name	Mechanism	References
circ-CHI3L1.2	Increases resistance to cisplatin through the miR-340-5p/LPAAT axis	[27]
hsa_circ_0004674	Regulates the miR-342-3p and FBN1 axis to promote OS doxorubicin resistance	[33]
CircPVT1	Via the miR-24-3p and KLF8, encourages OS cell proliferation and chemoresistance	[28]
	TRIAP1 is regulated by miR-137, which in turn contributes to OS cells' doxorubicin resistance	[37]
	Regulates ABCB1 and plays a role in OS cells' resistance to doxorubicin and cisplatin	[29]
circUBAP2	Boosts SEMA6D expression. The Wnt/-catenin signaling pathway can be activated to increase cisplatin resistance	[30]
hsa_circ_0000073	OS cells are more resistant to methotrexate because of it by upregulating NRAS and sponging miR-145-5p and miR-151-3p	[40]
circ_0081001	when knockdown, methotrexate sensitivity is increased by controlling the miR-494-3p and TGM2 axis	[41]
CircDOCK1	Via the miR-339-3p and IGF1R axis, aids in osteogenic sarcoma tumorigenesis and resistance to cisplatin	[31]
CircITCH	Through the miR-524/RASSF6 axis, downregulation of circITCH encourages OS development and resistance to doxorubicin	[38]
Circular RNA LARP4	Sponging microRNA-424 increases OS chemosensitivity to cisplatin and doxorubicin	[32]
Circular RNA_ANKIB1	Via microRNA-26b-5p, it speeds up resistance to chemotherapies	[39]

### CircRNAs and cisplatin resistance of OS

The competitive endogenous RNA mechanism plays a major role in the biological functions of circRNAs in cells because it contains miRNA-binding sites [12, 26]. CircCHI3L1.2 was found to be elevated in cisplatin-resistant OS cells [27], and the miR-340-5p and LPAAT axis could be used to make circCHI3L1.2-deficient OS cells more sensitive to cisplatin-resistant OS. They found that miR-340-5p could bind to circ-CHI3L1.2. Moreover, miR-340-5p's target, LPAATβ, had less protein expression when circ-CHI3L1.2 was knocked down. Notably, the effect of circ-CHI3L1.2 knockdown was mitigated by the miR-340-5p inhibitor. According to their findings, the miR-340-5p-LPAAT axis was involved in circ-CHI3L1.2's contribution to cisplatin resistance. The effects of circ-CHI3L1.2 knockdown were partially reversed by miR-340-5p suppression, which suggested that there were additional downstream pathways besides the miR-340-5p-LPAAT axis. Other potential mechanisms should be explored in future studies. Numerous cancers have been linked to the oncogene CircPVT1. CircPVT1 was found to be up-regulated in OS tissues resistant to cisplatin, doxorubicin or methotrexate [28]. The targeting relationships of circPVT1/miR-24-3p and miR-24-3p/KLF8 were verified. CircPVT1 could act as a sponge of miR-24-3p. A further confirmation of the negative regulation between circPVT1 and miR-24-3p was observed in OS cells. This research found that the overexpression of miR-24-3p inhibited the proliferation of OS cells, and increased the sensitivity of chemoresistant U2OS and MG63 cells to chemotherapy. The bioinformatics analysis suggested that KLF8 might be a downstream target of miR-24-3p. The binding association between miR-24-3p and KLF8 was confirmed by dual-luciferase reporter and RNA pull-down assays. KLF8 transcription factor played a vital role in oncogenic transformation. Nevertheless, the existing literature lacks a thorough discussion of the oncogenic role of KLF8 and its underlying mechanism. KLF8 was highly expressed in OS cell lines and was even further upregulated in chemoresistant OS cells, as confirmed by qRT-PCR and Western blotting assessments. The expression of KLF8 exhibited a positive correlation with that of circPVT1, while it demonstrated a negative association with that of miR-24-3p. Collectively, through the axis of miR-24-3p and KLF8, circPVT1 promotes OS cell proliferation and drug resistance. CircPVT1 is involved in drug resistance in OS tumor cells through multiple pathways. The overexpression of circPVT1 is responsible for OS cell drug resistance to doxorubicin and cisplatin by controlling the ATP-binding box (ABC) transporter ABCB1 [29]. OS cells resistant to cisplatin show elevated expression levels of CircuUBAP2 and SEMA6D. By activating the Wnt/β-catenin signaling pathway through the miR-506-3p/SEMA6D axis, circUBAP2 increases OS resistance to cisplatin [30]. CircDOCK1 [31] encourages OS cells to become cisplatin-resistant via the miR-339-3p and IGF1R axes. The circRNA LARP4 increases OS chemotherapy sensitivity to cisplatin and doxorubicin by sponging microRNA-424 [32]. The competitive endogenous RNA mechanism of circRNAs contributes to the resistance of OS to chemotherapy and several other biological functions.

### CircRNAs and doxorubicin resistance of OS

By controlling miR-342-3p and FBN1, hsa_circ_0004674 [33] promotes resistance to doxorubicin through the Wnt/β-catenin pathway, suggesting that hsa_circ_0004674 could be a promising target for OS resistance. The researchers found high levels of hsa_circ_0004674 expression in osteosarcoma cells and tissues that were resistant to doxorubicin. OS tumors became more sensitive to doxorubicin when hsa_circ_0004674 was knocked out. Moreover, their study discovered that miR-342-3p was under expressed in the doxorubicin-resistant OS tissues and cells, inhibiting OS doxorubicin resistance. The anti-miR-342-3p reversal effect on si-hsa_circ_0004674 suggested that hsa_circ_0004674 modulated the doxorubicin resistance of OS by targeting miR-342-3p. Studies unveiled that miR-342-3p targeted FBN1, whose aberrant expression is linked to the malignant phenotype of several tumors, such as ovarian cancer [34] and papillary thyroid carcinoma [35]. The results showed that miR-342-3p increased resistance to doxorubicin through its interaction with FBN1. The research suggests that during the malignant progression of many cancers, the activity of the Wnt/β-catenin signaling pathway is significantly upregulated [36]. Related studies of osteosarcoma have shown that activation of the Wnt/β-catenin pathway is associated with chemoresistance, whereas inhibiting this pathway has been demonstrated to enhance chemotherapy sensitivity. Silencing hsa_circ_0004674 was found to inhibit the activity of the Wnt/β-catenin pathway. Further analysis revealed that hsa_circ_0004674 had a positive regulatory effect on the activity of the Wnt/β-catenin pathway through the miR-342-3p/FBN1 axis. It has been reported that circPVT1 [37] was concerned with OS cell drug resistance to doxorubicin by controlling TRIAP1 through miR-137. circPVT1 knockdown could boost doxorubicin sensitivity by inhibiting doxorubicin-caused proliferation and doxorubicin -induced apoptosis in doxorubicin-resistant osteosarcoma cells in vitro. The mechanical analysis revealed that circPVT1 functioned as a miR-137 sponge to regulate TRIAP1 expression. Furthermore, a mechanistic analysis confirmed that the miR-137 inhibitor was able to partially reverse the inhibitory effect of silencing circPVT1 on the TRIAP1 level in doxorubicin-resistant osteosarcoma cells. This validates the role of circPVT1 as a miR-137 sponge that upregulates TRIAP1 expression. Through the miR-524/RASSF6 axis, circITCH downregulation promotes OS development and doxorubicin resistance [38]. The interaction between circITCH, miR-524, and RASSF6 was confirmed through dual-luciferase reporter and RNA immunoprecipitation assays. By binding to microRNA-26B-5P and modulating EZH2, circular RNAANKIB1 promotes chemotherapy resistance in OS [39]. The expression of miR-26b-5p was suppressed in both OS tissues and cells, as well as doxorubicin-resistant OS tissues and cells, while the levels of circ_ANKIB1 and EZH2 were increased. Circ_ANKIB1 binds to miR-26b-5p. MiR-26b-5p directly targeted EZH2, and increasing the levels of EZH2 reversed the effect of elevated miR-26b-5p on doxorubicin-resistant cells. In vivo, silencing of circ_ANKIB1 suppressed the growth of doxorubicin-resistant OS cells.

### CircRNAs and methotrexate resistance of OS

It was discovered for the first time that hsa_circ_0000073 may enhance the proliferation, migration, invasion and methotrexate resistance of OS cells [40]. It was discovered that the expression of hsa_circ_0000073 was highly upregulated in both OS cells and tissues, which in turn led to poor OS survival. In order to determine whether hsa_circ_0000073 is involved in the competitive endogenous RNA model, predictions were made and it was observed that miR-145-5p and miR-151-3p directly bind to hsa_circ_0000073. At the same time, miR-145-5p and miR-151-3p exhibited a negative correlation with hsa_circ_0000073. miR-145-5p and miR-151-3p directly regulate NRAS. In OS cells, hsa_circ_0000073 upregulates NRAS by inhibiting miR-145-5p and miR-151-3p. According to their study, hsa_circ_0000073 may enhance the proliferation, migration and invasion of OS cells by directing the regulation of NRAS through miR-145-5p or miR-151-3p. The authors also hypothesized that methotrexate resistance in OS could be closely associated with the hsa_circ_0000073/miR-145-5p and miR-151-3p/NRAS axes. Circ_0081001 [41] has been implicated in regulating the sensitivity of OS cells to methotrexate by controlling the miR-494-3p/TGM2 axis. In methotrexate-resistant OS tissues and cells, expression levels of Circ_0081001 and TGM2 were upregulated while miR-494-3p was downregulated. Interference with Circ_0081001 resulted in increased cell sensitivity to methotrexate by promoting apoptosis and inhibiting cell viability and metastasis in vitro. Furthermore, a molecular sponge effect of circ_0081001 on miR-494-3p led to the upregulation of TGM2 level. Knockdown of circ_0081001 inhibited methotrexate resistance by upregulating miR-494-3p and downregulating TGM2. The downregulation of Circ_0081001 improved methotrexate sensitivity of OS in vivo.

## LncRNA mediated competitive endogenous RNA in chemoresistance of OS

LncRNAs are a class of RNA molecules with lengths ranging from 200 to 100,000 nucleotides. They regulate gene expression at various levels but do not encode proteins [42, 43]. Several lncRNAs have unusually high expression levels in cancer cells and can function as oncogenes or tumor suppressors, participating in the formation and spread of tumor cells [44]. They also contribute biologically to resistance to chemotherapy. Numerous studies have demonstrated a connection between chemotherapy resistance and changes in the expression levels of certain lncRNAs in OS tumor cells [9, 45] (Table 2). LncRNAs play key roles in drug resistance. Generally, the levels of lncRNAs involved in OS drug resistance increase through a competitive endogenous RNA mechanism [46–48].

**Table 2:** lncRNAs associated with osteosarcoma chemotherapy resistance

Name	Mechanism	References
LncRNA TTN-AS1	Via the miR-134-5p and MBTD1, controls drug resistance and apoptosis in OS cells	[49]
LncRNA KCNQ1OT1	In OS, inhibiting resistance by controlling LARP1 mediated by miR-129-5p by knocking down KCNQ1OT1	[50]
	OS cells become more sensitive to CDDP if KCNQ1OT1 is knocked out by increasing Kcnq1 expression through DNMT1	[51]
LncRNA MIR17HG	OS resistance to cisplatin is aided by the SP1, MIR17HG, and miR-130a-3p	[46]
LncRNA FENDRR	Reduces the levels of ABCB1 and ABCC1 in OS cells, making them more sensitive to doxorubicin	[52]
LncRNA HOTAIR	Bolsters OS cells' cisplatin resistance via the STAT3 Axis of miR-106a-5p	[47]
LncRNA SNHG16	Sponges miR-16 to increase ATG4B expression, which in turn increases cisplatin resistance	[48]
LncRNA NORAD	Regulates OS's sensitivity to drug resistance by targeting miR-410-3p	[53]
LncRNA ANRIL	Through targeted regulation of miR-125a-5p, OS cell sensitivity to cisplatin can be increased by inhibiting the expression of the lncRNA ANRIL	[54]
LncRNA-SARCC	Targets Hexokinase 2 to increase OS's sensitivity to cisplatin by inhibiting glycolysis through miR-143	[55]
LncRNA MSC-AS1	By binding to microRNA-142, downregulated lncRNA MSC-AS1 increases cisplatin sensitivity	[56]
LncRNA OIP5-AS1	Sponging the miR-340-5p induces the LPAAT, PI3K, AKT, and mTOR signaling pathway, resulting in OS cisplatin resistance	[57]
	Through the miR-377-3p/FOSL2 axis, knockdown enhances cisplatin sensitivity in OS	[58]
LncRNA ROR	Sponges miR-153-3p to induce cisplatin resistance in OS	[59]
LncRNA NCK-AS1	In OS cells, inhibiting the lncRNA NCK-AS1 modifies miR-137 to control cisplatin resistance	[60]
LncRNA DNAJC3-AS1	Decreases OS's sensitivity to cisplatin, which was reversed by down-regulating DNAJC3, its sense-cognate gene	[61]
LncRNA HOTTIP	By activating the Wnt/-catenin pathway, lncRNA HOTTIP overexpression increases osteosarcoma cell chemoresistance	[62]

The researchers have confirmed that the lncRNA TTN-AS1 controls OS cell growth, apoptosis, and cisplatin resistance and promotes MBTD1 expression by targeting miR-134-5p [49]. Patients with OS showed high levels of lncRNA expression. Drug resistance can also be reduced by downregulating TTN-AS1. Zhang et al. [50] found that the lncRNA KCNQ1OT1 was expressed in the tumors and adjacent tissues of 30 patients with OS. The lncRNA KCNQ1OT1 inhibited miR-129-5p expression, which in turn promoted cell proliferation, invasion, drug resistance, and LARP1 expression. DNMT1-mediated Kcnq1 expression increases with the knock-out of KCNQ1OT1, making OS cells more sensitive to cisplatin [51]. By focusing on the miR-130a-3p/SP1 axis, MIR17HG helps OS cells develop cisplatin resistance [46]. Doxorubicin-resistant OS cells and tissues had lower levels of the lncRNA FENDRR, which was linked to worse prognoses in patients with OS [52]. The lncRNA HOTAIR is upregulated in cisplatin-resistant OS tumor cells [47]. By directly binding to and controlling miR-106a-5p, HOTAIR overexpression upregulates STAT3 expression, which is reduced in OS tissues and cisplatin-resistant cells. Cisplatin resistance and drug resistance-related gene expression in Saos2/cisplatin, MG-63/cisplatin, and U2-OS/cisplatin cells were diminished when HOTAIR was knocked down. OS tissues had considerably increased SNHG16 and ATG4B expression. A higher level of SNHG16 expression is linked to a worse prognosis in patients with OS [48].

In OS-resistant HOS/cisplatin cells, one study found that the expression of lncRNA NORAD and MRP1 mRNA and protein was significantly elevated, while the expression levels of miR-410-3p were considerably minimized [53]. When the lncRNA ANRIL was knocked down in U2-OS and Saos-2 OS cells, ANRIL-silenced cells became more susceptible to cisplatin [54]. In ANRIL-silenced cells, the level of miR-125a-5p, which binds to ANRIL, increased. Furthermore, there was a decrease in the expression of STAT3, which is a target of miR-125a-5p. The researchers demonstrated that by selectively regulating miR-125a-5p, sensitivity of OS cells to cisplatin was enhanced by suppressing lncRNA ANRIL expression. Wen et al. discovered that OS cells became cisplatin-sensitive when the lncRNA-SARCC was overexpressed [55]. Using microarray analysis, the authors found that SARCC increased miR-143 expression in OS. In contrast, SARCC and miR-143 expression were downregulated in cisplatin-resistant OS cells, making them resistant to cisplatin. In OS, miR-143 directly targets hexokinase 2 (HK2), the key enzyme in glycolysis. The RNA network SARCC-miR-143-HK may regulate OS chemosensitivity. By sponging miR-140-5p, the lncRNA MSC-AS1 triggers osteogenic differentiation [56]. In addition, when MSC-AS1 was silenced, cisplatin became more toxic to OS cells, and overexpression of MSC-AS1 in OS patients led to a worse prognosis. Increasing miR-142 to decrease CDK6 and deactivate the PI3K/AKT axis inhibited OS cell processes in tumor cells with silenced MSC-AS1. Previous research indicated significantly increased expression of the lncRNA OIP5-AS1 in cisplatin-resistant OS cells, leading to resistance through LPAAT, PI3K, AKT, and mTOR pathways [57]. Knocking out OIP5-AS1 effectively reduced cisplatin resistance. Knockdown of OIP5-AS1 enhanced cisplatin sensitivity in OS via the miR-377-3p and FOSL2 axes [58], while the lncRNA ROR [59] mediated cisplatin resistance in OS by controlling ABCB1 through miR-153-3p. NCK1-AS1 silencing restrained OS cell proliferation, migration, and invasion, and heightened their cisplatin sensitivity [60]. Cisplatin-resistant OS cells exhibited notable upregulation of lncRNA NCK1-AS1. Overexpressing miR-137 increased OS cells' sensitivity to cisplatin, but the effects were counteracted by high levels of NCK1-AS1 in cisplatin-resistant cells. Another study discovered elevated expression of the lncRNA DNAJC3-AS1 [61] in OS, decreasing OS sensitivity to cisplatin through a mechanistic process, which was reversed by downregulating the sense-cognate gene DNAJC3. Elevated lncRNA HOTTIP [62] promoted chemoresistance in OS by activating the Wnt/-catenin pathway.

In summary, understanding how changes in the expression levels of lncRNAs in OS tumor cells is related to chemotherapy resistance may help in selecting and developing effective therapeutics to overcome drug resistance, thereby improving OS treatment outcomes.

## Prospects

In addition to the molecular mechanisms discussed here, other factors influence drug resistance in OS, such as microenvironmental damage and cell cycle- or apoptosis-related mechanisms of chemotherapy resistance. To develop more effective treatments, it is essential to conduct in-depth research into the molecular mechanisms underlying OS resistance. This is important for reducing the incidence of OS resistance, improving the efficacy and prognosis of chemotherapy for OS, and developing better treatments. Numerous mechanisms and factors are involved in the development of resistance to tumor medication. It is difficult to identify a single mechanism that fully explains chemotherapeutic drug resistance. Many factors influence drug resistance in OS, including the effects of ncRNAs, ABC transporters, cancer stem cells, DNA repair factors, autophagy, microenvironmental damage, and cell cycle and apoptosis-related mechanisms of chemotherapy resistance. New approaches to cancer treatment, such as new anticancer medications, utilization of multifunctional nanocarriers, and RNA interference therapy [63], have emerged in light of the aforementioned mechanisms.

### Combination of at least two antitumor medications: increased lethality against tumor cells

Chemotherapy of osteosarcoma has evolved from the initial single-drug application to the current multidrug combination. The combination of a variety of chemotherapy drugs achieved a good therapeutic effect. At the same time, the efficacy and adverse reactions of the drug should be comprehensively considered when the combination of chemotherapy drugs is used. The adverse reactions should be minimized, and personalized treatment plans should be provided for patients. How to combine drugs reasonably become the future research direction of molecular targeted drugs.

### Prolonging the exposure to chemotherapeutic drugs

The toxicity of a drug to normal tissue limits the potential dose, and the kinetics of the drug (including absorptive capacity, in vivo distribution, metabolism, and elimination) limit the concentration at which the drug can reach the tumor tissue. The recent advancements in cancer nanotechnology can offer chemotherapeutic drugs longer exposure times and extended circulating half-lives. Nevertheless, the most fundamental solution is to prevent and overcome multiple-drug resistance to chemotherapy drugs.

### Build drug-resistant tumor animal models to test clinical relevance

Before the stage of clinical and translational medicine research, it is necessary to combine in vivo pharmacology methods and genomics analysis platforms. Through the creation of animal models with drug-resistant tumors, the mechanism behind OS resistance can be better understood, and an optimal dosage regimen can be established. This approach can lead to the design of more effective OS drugs that demonstrate clinical benefits, ultimately fostering a new generation of anticancer medications. The establishment of dependable preclinical tumor drug-resistant cell models holds significant importance for delving further into the attributes of drug-resistant cells and for identifying novel clinical therapeutic strategies.

### Establish highly selected and annotated databases of germline and somatic mutant genes

Through genetic testing, the type of genetic variation and level of biomarkers in a patient's body can be determined, and clinicians can make decisions based on a comprehensive analysis of these indicators. The established databases include mutation points associated with drug responses, providing a description of the possible role of gene mutation sites in drug resistance and the level of evidence to effectively address possible drug intolerance or toxic side effects in patients with cancer.

With the discovery, prediction, and clinical application of molecular diagnostic markers, precise treatments for different patients are also being promoted. The drug resistance of tumor cells can be viewed from an evolutionary perspective, and a variety of therapeutic methods used to combat tumor cells. The most important challenge in tumor resistance is to quickly identify biological indicators of multiple-drug resistance before the emergence of tumor resistance. Through high-throughput screening techniques and systems biology approaches, researchers can partially detect or predict the response of tumor cells to particular chemotherapeutic drugs, thus avoiding complications before administering chemotherapy. In the future, the research direction of tumor resistance may be to identify molecular markers of tumor resistance, predict and monitor chemotherapy efficacy, perform early detection and prognosis combined with laboratory and imaging examinations, and develop chemotherapy drugs for effective targets.

## Conclusion

In this review, we discussed the molecular mechanisms underlying chemotherapy resistance in OS, especially in terms of circRNA-, lncRNA-, and miRNA-mediated competitive endogenous networks. CircRNAs bind to miRNAs and act as miRNA sponges, thus regulating target genes of miRNAs [61]. This is known as competitive endogenous RNA mechanism. Through their interactions with miRNAs, circRNAs play an important regulatory role in tumorigenesis and tumor progression. The researchers have found abnormal expression of circRNAs/lncRNAs in drug-resistant OS cells, suggesting that circRNAs/lncRNAs play a role in chemotherapy resistance in OS. The role of circRNAs/lncRNAs has also been explored. These findings provide a foundation for elucidating the mechanism of cisplatin resistance in OS and, eventually, new intervention targets for ncRNA-based therapeutics in OS, with the goal of preventing chemoresistance. The implications are significant, both in terms of advancing oncology research and actual patient outcomes. However, the research on the molecular mechanisms underlying chemotherapeutic drug resistance in OS is still in the early stages of development. Further studies are required to elucidate the involvement of ncRNAs in drug resistance of OS.

## Data Availability

Not applicable.

## Abbreviations

OS: Osteosarcoma
ncRNAs: Noncoding RNAs
lncRNA: Long noncoding RNA
circRNA: Circle RNA
miRNAs: Micro RNAs
mRNA: Messenger RNA
LPAATβ: Lysophosphatidic acid acyltransferase
MBTD1: Malignant brain tumor domain 1
ABC: ATP-binding cassette transporter
ABCB1: ATP-binding cassette subfamily B member 1
SEMA6D: Semaphorins 6D
SNHG16: Small nucleolar RNA host gene 16
ATG4B: Autophagy-related 4B
